# Effect of Ampicillin on the kinetics of colonization of *Streptococcus pneumoniae *and *Lactobacillus fermentum *in the respiratory tract of mice

**DOI:** 10.1186/1476-0711-3-23

**Published:** 2004-10-27

**Authors:** Rosa Cangemi de Gutiérrez, Viviana Santos, Marta Cecilia, Clara Silva, María Elena Nader-Macías

**Affiliations:** 1 Facultad de Bioquímica, Química y Farmacia. Universidad Nacional de Tucumán. Tucumán. Argentina; 2 CERELA-CONICET. Chacabuco 145. 4000. Tucumán. Argentina

**Keywords:** *S. pneumoniae*, *L. fermentum*, probiotics, Ampicillin, antimicrobials, colonization

## Abstract

Ampicillin was selected to further study the effect of this antibiotic on the colonization capability of *S. pneumoniae *and *L. fermentum *intranasally inoculated in a mice experimental model. The sensitivity of *S. pneumoniae *and *L. fermentum *to antibiotics was evaluated by different "in vitro" techniques. The results showed that both microorganisms have a typical pattern of sensitivity to antibiotics in these assays. The "in vivo" experiments showed that the treatment with Ampicillin increased the number of lactobacilli and *neumococci *in the groups of mice treated only with one of the microorganisms. In those mice treated with *Lactobacillus*, challenged later with *neumococci *and treated with Ampicillin, the pathogen in lung decreased on the 4^th ^day, disappearing completely after on. The histological studies showed that the antibiotic treatment decreased the inflammatory response produced by the pathogen at the lung and trachea levels.

## Introduction

Respiratory tract infections are commonly caused by *Streptococcus pneumoniae*. Extensive antibiotic use for these infections as well as misuse for viral respiratory infections has led to increased penicillin resistance amongst the streptococci [1-6]. Even though there is a very broad description of the pattern of sensibility to antibiotics of this pathogen and other potentially pathogenic microorganisms, there are a small number of publications referred to the lactobacilli sensitivity to these types of compounds [7-10]. The antibiotic treatment modifies the stability of the normal or indigenous microbiota, producing the dominance of certain microorganisms able sometimes to produce a secondary infection [11]. There are a lot of approaches trying to restore the normal microbiota, or to avoid modifications of the different ecosystems to prevent infections, both for human and animal application. One of the main research areas related to the restoration of the indigenous flora is the application of probiotic microorganisms [12-14].

Lactic Acid Bacteria (LAB) has been widely used to restore the ecologic equilibrium of different areas [12-14], mainly for the gastrointestinal tract. In the last decade, there is a lot of other research areas referred to the potential application of probiotics in the respiratory tract mainly as vaccine vectors [15-17]. They could be applied for the protection against pathogenic microorganisms as *S. pneumoniae *which is a frequent nasopharynx colonizer. In previous papers, the isolation and identification of the microorganisms of the normal microbiota of the respiratory tract of mice was reported by our research group. Also, the evolution from the moment they were born up to two months age was published [18]. In the isolated microorganisms, the probiotic or beneficial characteristics were studied, selecting some strains of the genus *Lactobacillus *that shared some properties [19]. From them, a strain of *Lactobacillus fermentum *was selected by the beneficial probiotic properties. Later the optimal dose to produce a transitory colonization or permanence [20,21] and the protection exerted against *S. pneumoniae *[22] intranasally inoculated was determined.

The objective of the present paper was to study the effect of specific antimicrobial agents against the growth of *S. pneumoniae *and *L. fermentum *by "in vitro" assays. Also, to study the effect of Ampicillin, orally administered in a mice experimental model, against the intranasal inoculation of *S. pneumoniae*, *L. fermentum *or both microorganisms. The "in vivo" assays were complemented with histological and cytological studies to determine if there was some type of general response of the animals to the treatment.

## Materials and methods

### Microorganisms and culture media

*L. fermentum *was isolated from the respiratory tract (pharynx) of adult BALB/c mice [18,20-22]. The mutants resistant to Rifampicin (RR) were obtained to differentiate the inoculated strains from the normal microbiota. The conditions of storage and culture were described previously [18,20-22]. *S. pneumoniae *A6 serotype was isolated from human pneumonia-suffering subjects, and identified by standard techniques. The serotypification was performed at the "Servicio de Bacteriología Clínica, Instituto Nacional de Enfermedades Infecciosas-ANLIS "Dr Carlos G. Malbran, Buenos Aires, Argentina by Quellung technique. Pathogenicity in mice was increased by inoculating *S. pneumoniae *intraperitoneally [22]. The pathogen was stored in 25% glycerol added to BHI broth (Brain-Heart Infusion) at -70°C.

### Antibiotics assayed

those antibiotics broadly used for the treatment of Gram Positive microorganisms affecting the respiratory tract were studied by determining the Minimal Inhibitory Concentration: penicillin, ampicillin, ceftriazone, ceftazidine, claritromicine, tetracicline, rifampicin, ciprofloxacin, aztreonam, cloramphenicol and imipenen.

### Quantitative determination of the bactericidal activity of antibiotics against lactobacilli

The Minimal Inhibitory Concentration Method (MIC) recommended by the National Committee for Clinical Laboratory Standards (NCCLS) was used, replacing the culture media by MRS agar as basal media [23], because Lactic acid bacteria are not able to grow in the recommended Muller Hinton agar. The reference strains used were *Enterococcus faecalis ATCC 29212 *and *Staphylococcus aureus ATCC 29213*.

### Antibiotic sensitivity of *S. pneumoniae*. Method of diffusion and dilution in agar

The behavior of the pathogenic microorganisms with antimicrobial substances was determined by the qualitative and quantitative techniques of diffusion and dilution in agar (MIC), according to the National Committee for Clinical Laboratory Standards, 2001 (NCCLS). The antibiotics assayed for the qualitative method were oxacillin, vancomycin, chloramphenicol, tetracycline, rifampicin, ciprofloxacin, trimetroprim-sulphametoxazol, claritromycin. The antibiotics assayed for the quantitative method were penicillin, ampicillin, ceftriazone and claritromycin. The reference strain used was *S. pneumoniae *ATCC 49619.

### "In vivo" assays

Each experimental group (or assay) included 24 to 30 animals. Four to six mice were killed in each experimental day. Each one of the "in vivo" experiments was performed twice, and their data used to calculate the media and Standard Deviation. The protocols used were accepted by the Animal Ethics committee of CERELA.

### *L. fermentum *and Ampicillin assay

**Figure 1:**
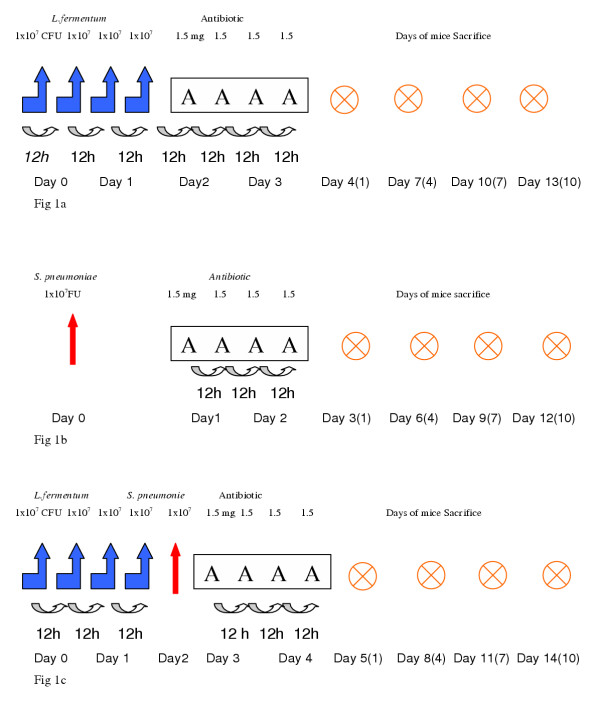
Experimental protocols, doses of microorganism and time schedule applied to perform the "in vivo" assays: **Fig 1a: **Administration of *L. fermentum *(empty arrows) and Ampicillin (A). **Fig 1b**: Administration of *S. pneumoniae *(red arrow) and Ampicillin (A). **Fig 1c: **Administration of *L. fermentum*, *S. pneumoniae *and Ampicillin. The days when mice were sacrificed are indicated with black crosses.

groups of adult (two months old) male BALB/c mice were intranasally inoculated with 4 doses of a RR *L. fermentum *strain every 12 h. (50 μl of a suspension containing 1 × 10^9 ^CFU/ml). Later, Ampicillin was administered by the oral way in 4 doses of 100 mg/Kg/day, fractionated in two daily doses (1.5 mg/dose/mice) every 12 h. The experimental protocol is schematized in Fig 1a.

### *S. pneumoniae *and Ampicillin assay

BALB/c mice were intranasally inoculated with a dose of 50 μl of a suspension of *S. pneumoniae *in saline solution (1 × 10^9 ^CFU/ml). The antibiotic was administered by the oral way in 4 doses of 100 mg/Kg/day, following the same scheme than before. The experimental protocol is schematized in Fig 1b.

### *L. fermentum *+ *S. pneumoniae *+ Ampicilin assay

Mice were intranasally inoculated with RR *L. fermentum *(4 doses of 50 μl of a suspension of 1 × 10^9 ^CFU/ml), then challenged with *S. pneumoniae *A6 by the same way (50 μl of a suspension of 1 × 10^9 ^CFU/ml) and later treated with Ampicillin in four doses every 12 hours as before, as shown in Fig 1c.

All the experimental groups were sacrificed by cervical dislocation on days 2, 4, 7 and 10 post antibiotic administrations. The samples from nasopharynx and pharynx were obtained with a cotton swab. Trachea, bronchia and lung were aseptically removed and homogenized with a Teflon pestle. The number of microorganisms was determined by the successive dilutions method with peptone water, and plated in MRS agar-Rifampicin. The plates were incubated for 24 h. at 37°C in microaerophilic environment. The identification of microorganisms was performed by macroscopic, microscopic characteristics, Gram staining and biochemical tests.

### Histological studies

the samples for histological assays were obtained from the higher trachea, located at the neck basis, bronchia in the area where the main bronchia are divided, and lung in the terminal bronchiole area and alveolar wall. They were fixed with 10% paraformaldehyde and stained with Hematoxylin-eosin and Ramon and Cajal technique [24]. They were analyzed by light microscopy.

### Cytological technique

The left lobule of the lung was used to perform the cytological slices. The organ was first immersed in saline solution, then fixed in methanol for 3 min, and afterwards stained with the Romanovsky method (Giemsa stain-Merck,Germany) used routinely in the lab [25].

### Statistical techniques

The numbers in the figures represent the mean and Standard Deviation of the results obtained in the two set of experiments performed for each assay. The Student's t test was applied to determine the differences statistically significant of the data.

## Results

### Antimicrobial sensitivity

#### *L. fermentum*

The MIC results for *L. fermentum *(ug/ml) are as follows: penicillin, ampicilin, ceftriazone, ceftazidine, rifampicin, ciprofloxacin: ≥100 ug/ml, tetraciclin, imipenem and aztreonam: 10 ug/ml, cloramphenicol 1 ug/ml, claritromicine: 0.1 ug/ml. Comparing these values with those obtained for the MIC of *Enterococcus spp *and *Streptococcus spp*, type strains recommended by NCCLS, *L. fermentum *would be resistant to penicillin, ampicillin, ceftriazone, rifampicin and ciprofloxacin.

#### *S. pneumoniae*

The results of the antimicrobial sensibility of *S. pneumoniae *are shown in Table 1 and 2, demonstrating by the two methods applied that *S. pneumoniae *is sensitive to all the antibiotics assayed by both, the disc diffusion assay, and the MIC.

**Table 1:** Sensitivity of *S. pneumoniae *to different antimicrobials by the disc diffusion assay

**Antibiotic**	**Concentration (ug)**	**Diameter (mm)**		**Results**
		***R^(1)^***	***S^(2)^***	
**Oxacillin**	1	-	≥20	24
**Vancomycin**	30	-	≥17	22
**Chloramphenicol**	30	≤20	≥21	24
**Tetracycline**	30	≤18	≥23	32
**Rifampicin**	5	≤16	≥19	28
**Ciprofloxacin**	5	≤13	≥17	29
**Trimetoprim+Sulphametoxazol**	1.25 + 23.75	≤15	≥19	26
**Claritromicin**	15	≤16	≥21	25

(1) Resistant, (2) Sensitive.

**Table 2:** Sensitivity of *S. pneumoniae *to antimicrobials by the Agar dilution method (MIC)

**Antibiotic**	**MIC (ug/ml) Break point**	**Sensitive**	**Results (ug/ml)**
**Penicillin**	-	≤0.12	≤0.02
**Ampicillin**	-	≤0.25	≤0.10
**Ceftriazone**	≥2	≤0.50	≤0.10
**Claritromicin**	≥1	≤0.25	≤0.02

#### "In vivo" assays

from the results obtained in "in vitro" sensitivity assays, that are predictive, the antibiotic Ampicillin was selected to be used in "*in vivo*" assays at the recommended dose for human beings (equivalent to the one applied to respiratory tract infections therapy).

#### Mice treated with *L. fermentum *(1 × 10^7 ^CFU/ml/dose) and Ampicillin

**Figure 2:**
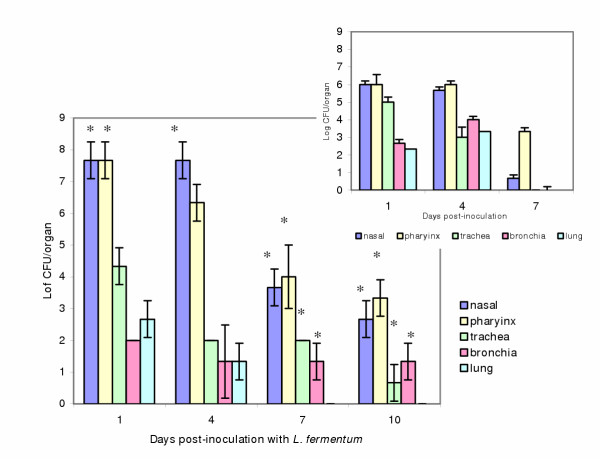
Colonization of *L. fermentum *in the respiratory tract of mice obtained after the administration of Ampicillin. The figures express the number of *L. fermentum *in nasal instillations, pharynx, trachea, bronchia or lung from mice inoculated intranasally with *L. fermentum *(4 doses of 10^7 ^CFU/mouse) and treated with 4 doses of Ampicillin. The inserted figure shows the number of lactobacilli obtained from control mice without antibiotic. (*) indicates differences statistically significant between the two groups of mice

Mice inoculated intranasally with four doses of lactobacilli (determined previously as the dose needed to obtain colonization of the respiratory tract) and treated with Ampicillin showed an increased colonization of lactobacilli during all the days studied in nasal and pharynx exudates (p < 0.01), with values of 10^7–8 ^CFU/organ on days 1 and 4, and lower values on the following days. The higher number were in nasal and pharynx exudates. In the control mice without antibiotic, *Lactobacillus *numbers were around 10^6 ^CFU on the first days of the assay. On the 7^th ^and 10^th ^days with antibiotic treatment, *Lactobacillus *were still present in all the organs, except in lung, while in the mice without antibiotics they were only present on nasal and pharynx exudates on day 7 disappearing on day 10 (upper right Figure 2). The results obtained are shown in Figure 2.

#### Mice challenged with *S. pneumoniae *(1 × 10^7 ^CFU/ml) and treated with Ampicillin

**Figure 3:**
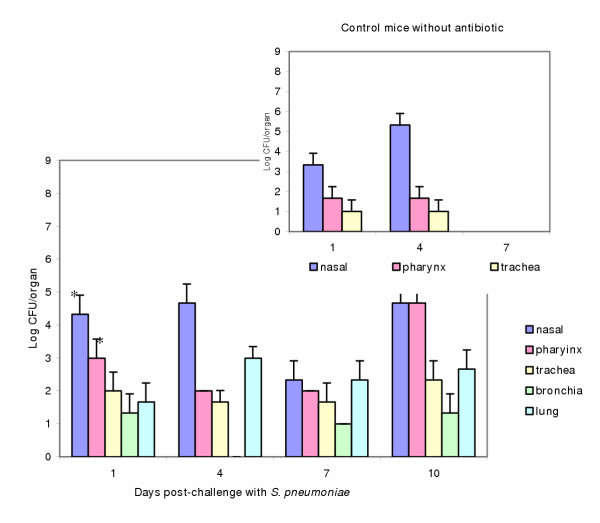
Colonization of *S. pneumoniae *in the respiratory tract of mice obtained after the administration of Ampicillin. The figures express the number of *S. pneumoniae *in nasal instillations, pharynx, trachea, bronchia or lung from mice, challenged with *S. pneumoniae *(10^8 ^CFU/mouse) and treated with 4 doses of Ampicillin. The inserted figure shows the number of pathogens obtained from control mice without antibiotic. (*) indicates differences statistically significant between the two groups of mice

Mice challenged with the pathogen and treated with the antibiotic showed that *neumococci *were present a longer time in all the organs of the respiratory tract, (as shown in Figure 3) compared with the respective mice without antibiotic treatment (upper right figure 3). The differences statistically significant (p < 0.01) between the two groups of mice are indicated in the figure.

#### Mice treated with *L. fermentum*, *S. pneumoniae *and Ampicillin

**Figure 4:**
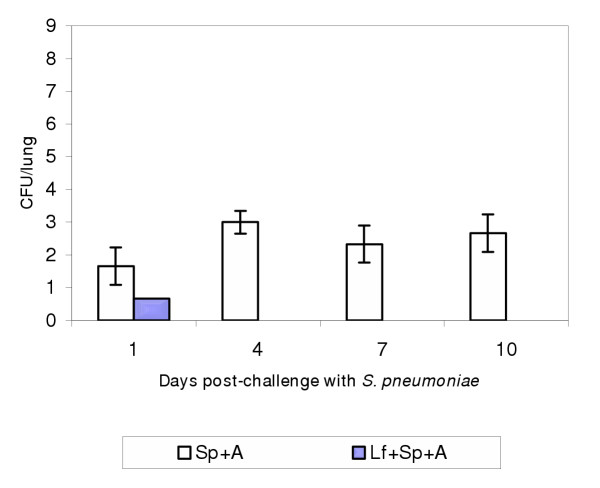
Colonization *S. pneumoniae *in mice inoculated intranasally with *L. fermentum *(4 doses of 1 × 10^7 ^CFU/dose), challenged with *S. pneumoniae *(1 × 10^8 ^CFU/mice) and treated with 4 doses of Ampicillin compared with mice treated only with the pathogen and antibiotics. The figures express the number of pathogen in lung.

The colonization of *Lactobacillus *in this group was similar to the one obtained in the experimental group treated with *Lactobacillus *and antibiotics without the pathogen (not showed results) In the case of *S. pneumoniae*, the previous treatment with *Lactobacillus*, reduced significantly the number of pathogenic microorganism in the lung, as showed in Fig 4. It was present only on the first day, being completely cleared on the following days studied.

#### Histological and cytological studies

**Figure 5:**
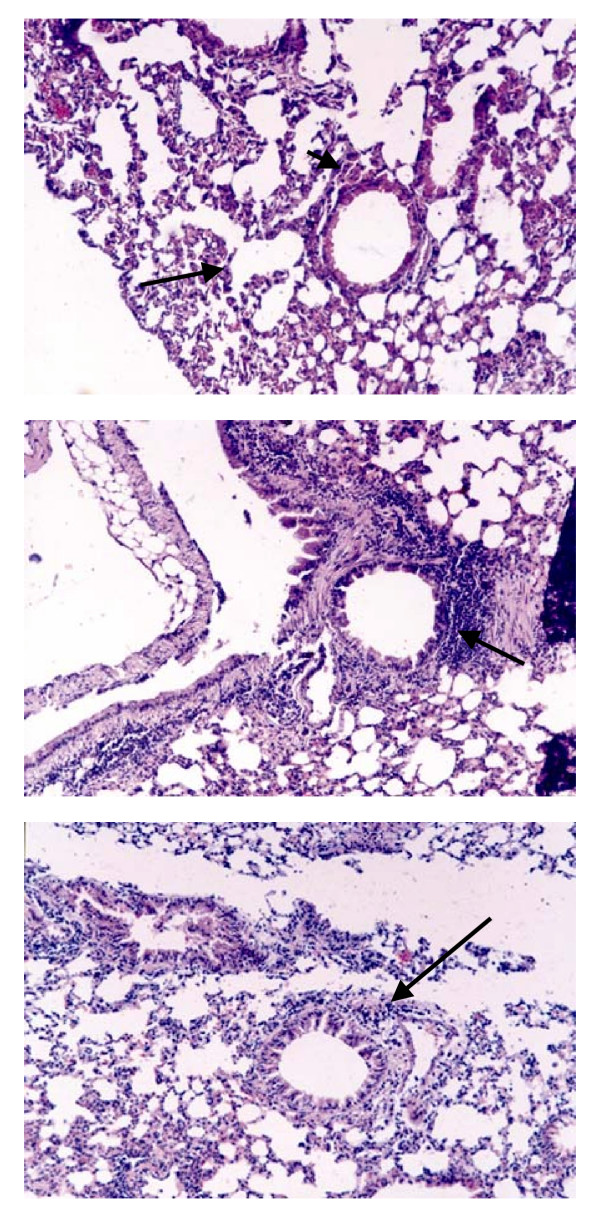
Light microscopy photographs (200×) of histological slices stained with hematoxylin-eosin from mice intranasally treated with *L. fermentum*, (5a) *S. pneumoniae *(5b) or both (5c) and later with Ampicillin. Fig 5a: regular ducts (long arrow) and alveolus (short arrow) (100×), Fig 5b: regular mononuclear exudation close to the interalveolar duct (100×). Fig 5c: Increased lymphocytic density (100×).

The histological studies showed that in all the organs of the respiratory tract there are not significant structural modifications. Only a moderate leukocyte exudation is observed in the alveolar region in the mice treated with *S. pneumoniae a*nd Ampicillin These results are resumed in Table 3. The administration of *Lactobacillus *produces a stimulation of the lung macrophages, and a lymphocytic infiltration at the trachea level, as resumed in Table 3 and Figure 5a. Mice treated with lactobacilli, *S. pneumoniae *and antibiotic decreased the inflammatory response produced by the pathogen (picture 5b) compared with the pattern produced by the pathogen and antibiotic, represented in Fig 5c.

**Table 3:** Histological modifications of the respiratory tract of mice on day 4 of the experiment.

**Organ**	**control**	***L.f*.+ampicillin^(1)^**	***S.p*.+ampicillin^(2)^**	***L.f*.+*S.p*.+ampicillin^(3)^**
Bronchia	Cilindric epithelia	Conserved	Conserved	Conserved
Bronchioli	Cubic epithelia	Conserved	Without extension of the interalveolar wall	Conserved
Lungs	Alveolus and duct with plane epithelia.	Regular duct and alveolus. Alveolar light with macrophages	Regular exudation of mononuclear lymphocytes close to the interaveolar duct.	Slight areas with a scarce increased lymphocytic density

Mice were inoculated with: *L. fermentum *+ ampicillin (1), *S. pneumoniae *+ ampicillin (2) and *L. fermentum *+ *S. pneumoniae *+ ampicillin (3).

The cytological studies showed an increased number of activated macrophages by *Lactobacillus*, *neumococci*, or both. The addition of the antibiotic produces an activation of the non-specific line of defense represented by the alveolar macrophages, as shown in Table 4.

**Table 4:** Cytology of lung impressions stained with Giemsa on day 4 post inoculation in mice treated with *L. fermentum*+*S. pneumoniae*+ampicillin

	**Normal Macrophages**	**G1 Macrophages**	**G2 Macrophages**	**Lymphocytes**	**Neutrophils**	**Other cells**
**Control Mice**	26 +/- 2	13 +/- 4	10 +/- 2	25 +/- 4	9 +/- 3	17 +/- 5
***L. fermentum *+ ampicillin**	14 +/- 4	30 +/- 5	28 +/- 4	14 +/- 3	9 +/- 2	5 +/- 4
***S. pneumoniae*+ampicillin**	18 +/- 3	25 +/- 5	30 +/- 2	7 +/- 4	12 +/- 2	8 +/- 5
***L.f *+ *S.p*. + ampicillin**	20 +/- 2	30 +/- 2	25 +/- 3	13 +/- 2	6 +/- 2	6 +/- 3

## Discussion

One of the main goals of our research is to go further into the mechanisms involved in the probiotic effect in the respiratory tract [26,27]. Having in mind that we have available a mice experimental model, our interest was focused in the knowledge of the effect produced by the antibiotics more frequently used to treat the respiratory infections on the kinetics of colonization of both microorganisms, either separately or combined. We were also interested in the effect produced by antibiotics on the microbial colonization by the potential use of probiotics together with antibiotics as therapeutic agents and for the restoration of the normal microbiota.

Our results showed that the administration of Ampicillin by the oral route and *Lactobacilus *intranasally increases their colonization in the respiratory tract. These results support the use of antibiotics together with probiotics, which would help in having a higher colonization of the protective microorganisms. A paper published by Dielemen et al [28] demonstrated that the administration of Lactobacillus GG prevents recurrence of colitis in transgenic rats after the treatment with Vancomycin and Imipenem, showing that our hypothesis has been proved in the intestinal tract.

Coincidently with these results, the treatment with Ampicillin, even though the *S. pneumoniae *strain used was sensitive to Ampicillin in the "in vitro" test, in the experiments performed in mice produces an increase in the number of the pathogenic microorganism that are also present a longer time in the tract. The pathogen produces a lymphocytic infiltration al the tracheal level, together with an increased inflammatory response evidenced in the lung cytological studies.

Why the response to the pathogen is different in "in vitro" assays than in "in vivo" experiments?. Referred to the behavior of *Lactobacillus*, there is a broad discussion based on the different results obtained through the application of "in vitro" test, and the differences found in "in vivo" assays. Even though the first one can be used as screening and to predict some characteristics or properties assayed in experimental animals models, one must consider that not always they are coincident [29].

In those experiments performed in mice treated with *Lactobacillus*, neumococci and Ampicillin, the final results is that the pathogen was cleared faster from the lung supporting the combined use of lactobacilli and antibiotics in the prevention of the infections produced by this pathogen. These results are also demonstrated by the histological slides, (Fig 5c) where the effect produced by the pneumococci decreased by the concomitant use of lactobacilli and antibiotics, when compared with the damage produced by the pathogen administration (Fig 5b). The histological modifications produced by the pathogen in mice previously protected with lactobacilli is lower compared to that obtained when the pathogen alone is inoculated into mice, [20] which produces congestion zones and edema in the terminal bronchiolar area.

The activation of lung or alveolar macrophages produced by the administration of *L. fermentum*, *S. pneumoniae*, or both, indicates the highly active non-specific branch of the immune system, which is an important first line of defense against microbial invasion in the lower airways infection. This activation could also help in the clearance of the neumococci observed on the 4th day of the experiments. These results do not agree with those from one infections produced by *Pseudomonas aeruginosa*, that does not produce an activation of the Pulmonary Alveolar Macrophages [30]. The activation of the immune system at the respiratory tract is taking more relevance, mainly by some researchers who study this way of administration of different antigens and vaccines [15-17].

More studies must be undertaken trying to elucidate which are the mechanisms involved in the protection exerted by lactobacilli in the respiratory tract, and also which are the reasons of the increased colonization of the pathogen and lactobacilli by the effect of the Ampicillin treatment.

## Authors' contributions

**Rosa Cangemi de Gutiérrez, **carried out the experimental and microbiological assays in animals

**Viviana Santos, **performed the histological and cytological studies

**Marta Cecilia, **participated in the interpretation of antibiotic assays

**Clara Silva, **carried out the in vitro sensibility test

**María Elena Nader-Macías: **conceived the study and participated in its design, evaluation of the results and writing of the manuscript
